# Central vein stenosis in a dialysis patient: a case report

**DOI:** 10.1186/1752-1947-6-189

**Published:** 2012-07-09

**Authors:** Uwe Gottmann, Maliha Sadick, Kathrin Kleinhuber, Urs Benck, Kurt Huck, Bernhard K Krämer, Rainer Birck

**Affiliations:** 1 5th Department of Medicine (Nephrology/Endocrinology/Rheumatology), University Hospital Mannheim, University of Heidelberg, Theodor-Kutzer-Ufer 1-3, D-68135, Mannheim, Germany; 2 Institute of Clinical Radiology and Nuclear Medicine, University Hospital Mannheim, University of Heidelberg, Theodor-Kutzer-Ufer 1-3, D-68135, Mannheim, Germany; 3 1st Department of Medicine (Cardiology/Angiology/Pneumology), University Hospital Mannheim, University of Heidelberg, Theodor-Kutzer-Ufer 1-3, D-68135, Mannheim, Germany

## Abstract

**Introduction:**

Central vein stenosis is not a rare problem in patients on dialysis. Placement of a central vein catheter for dialysis access substantially increases the risk of central vein stenosis. However, even in patients without a previous history of central vein catheter placement, a stenosis can be found in up to 40% of patients.

**Case presentation:**

We report the case of a 60-year-old male Caucasian German dialysis patient who complained of dry cough, swelling of his right arm and facial edema. Computed tomography venography showed a near-total stenosis of his brachiocephalic vein. We discuss the incidence and risk of central vein stenosis in patients on dialysis and report on a successful minimally invasive interventional treatment.

**Conclusion:**

Central vein stenosis is not a rare problem in patients on hemodialysis and can even occur without previous placement of central venous catheters. High shunt volumes seem to increase the risk associated with central vein catheters.

## Introduction

Patients undergoing hemodialysis need chronic vascular access to provide repeated access to the circulation with minimal complications. Complications associated with vascular access include stenosis and thrombosis, infection, digital ischemia, heart failure, pseudoaneurysm and aneurysm. Central venous cannulation can lead to the development of central venous stenosis. In patients on dialysis, this is primarily related to the placement of an ipsilateral central venous catheter but can also occur without a previous history of catheter placement [1,2]. It is expected that central venous cannulation leads to intimal injury associated with focal endothelial denudation, increased smooth muscle cells [3] and vein wall thickening. The rapid blood flows associated with the hemodialysis catheter can create turbulence that accelerates endothelial proliferation, eventually leading to venous stenosis. Early placement of an arteriovenous access prior to initiating dialysis can reduce the need for central venous catheters and thus reduce the prevalence of central vein stenosis.

## Case presentation

We report the case of central vein stenosis in a 60-year-old male Caucasian German dialysis patient. Our patient became dialysis-dependent in the 1970s because of postinfectious glomerulonephritis. During a 40-year history of end-stage renal disease he underwent renal transplantation three times with several years of dialysis in-between. In the meantime, and after the failure of his last renal transplant, the insertion of numerous central vein catheters from both his internal jugular veins was necessary to provide temporary dialysis access during recurrent arteriovenous fistula problems. After the final failure of an autogenous left arm access, a transposed brachial-basilic access was created in his right arm.

**Figure 1:**
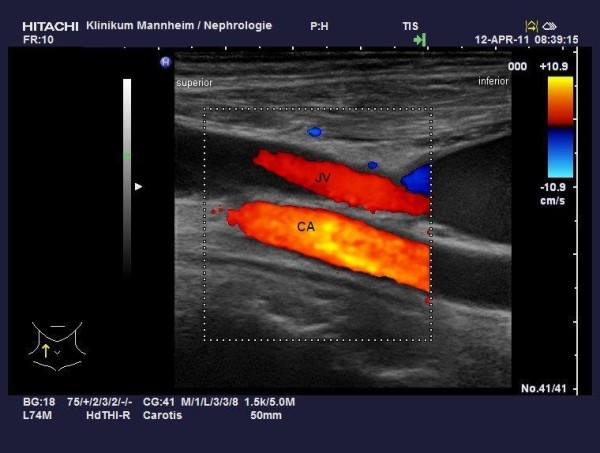
Color duplex sonography of the right jugular vein and carotid artery showing a cranial flow direction in both.

**Figure 2:**
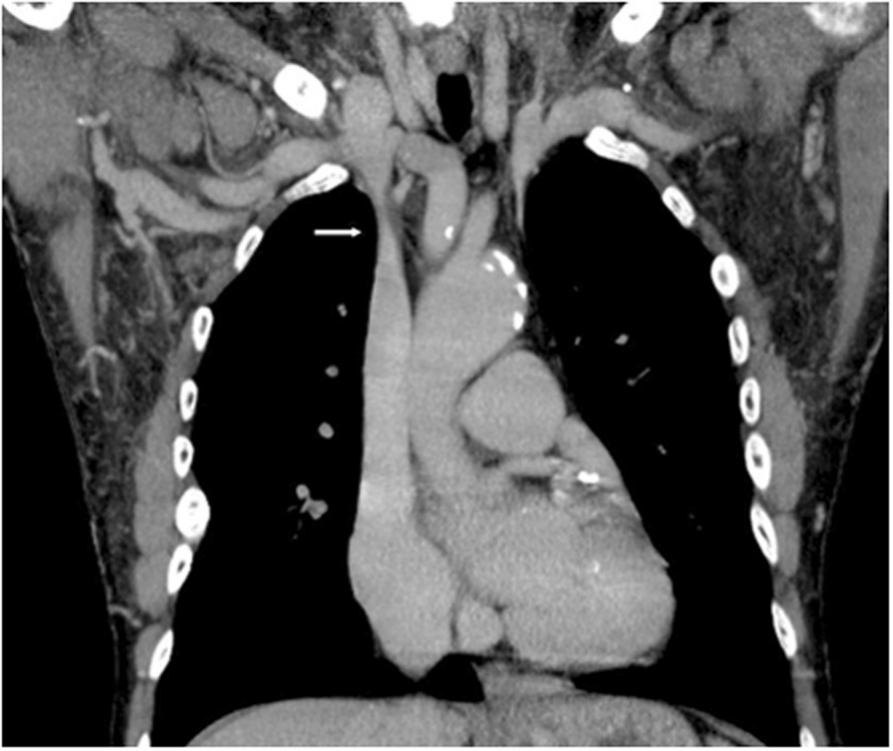
**Computed tomography venography, coronal view of the upper chest.** The high grade stenosis of the brachiocephalic vein is marked by an arrow.

**Figure 3:**
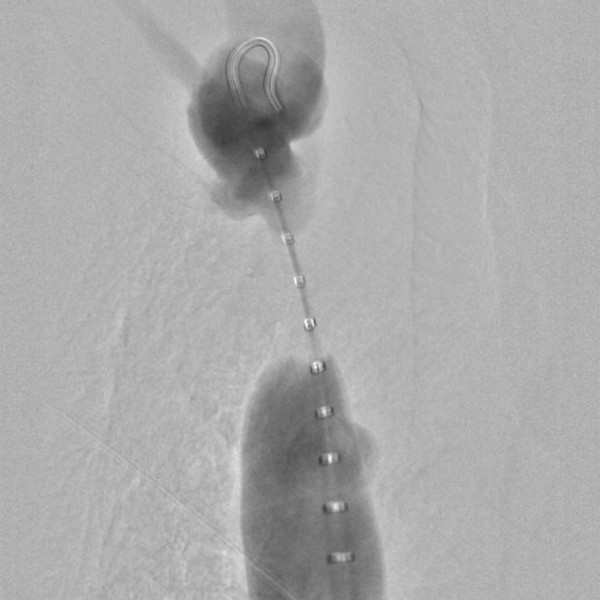
**Conventional central venogram demonstrating the 5 F Pigtail catheter in the superior vena cava.** A high grade stenosis, without contrast media passage through the stenosis, is visible.

**Figure 4:**
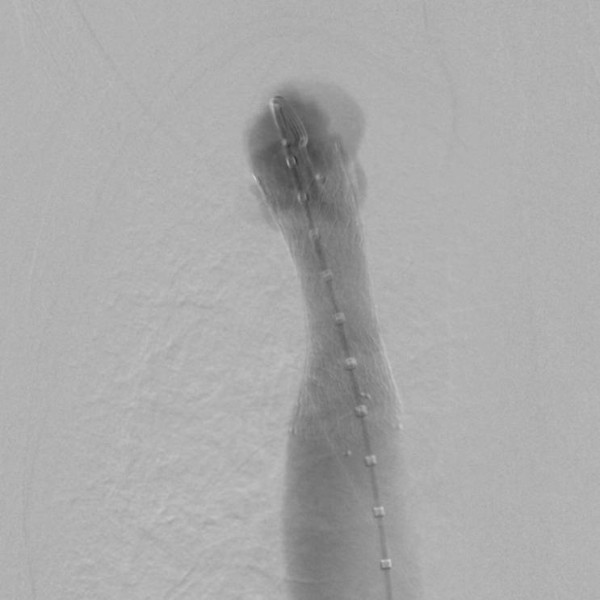
Conventional central venogram after balloon angioplasty and placement of a self-expanding stent (Sinus XL Stent 22/60mm) demonstrating an almost normal caliber of the brachiocephalic vein with contrast media flow through the stent lumen towards the heart.

Three years later, our patient reported swelling of his right arm and dry cough. In additional, a clinical investigation showed facial edema and jugular vein engorgement. Color Doppler sonography revealed a reversed flow in his jugular vein, in a cranial direction (Figure 1) and a turbulent flow in his brachiocephalic vein without clear delineation of a stenosis. Computed tomography venography coronal views of his neck and upper chest revealed subtotal stenosis of his right brachiocephalic vein (Figure 2) with collateral circulation from his internal jugular vein over the external jugular veins, sinus cavernosus, paravertrebral veins and the veins of the mediastinal cavity. Conventional catheter phlebography via a venous transfemoral access showed subtotal stenosis of his right brachiocephalic vein over a length of nearly 4cm (Figure 3). Subsequently, the stenosis was dilated by balloon angioplasty (MAXI balloon 12/40mm, Cordis®, Bridgewtaer, NJ, USA, and Atlas balloon 16/40mm, Bard®, Murray Hill, NJ, USA). Because of the acute elastic recoil of the vein, a self-expanding stent (Sinus XL Stent 22/60mm, Optimed®,Ettlingen, Germany) was placed with a good post interventional result (Figure 4).

Four weeks after stent implantation, our patient’s facial edema and jugular vein engorgement were resolved, and color Doppler sonography showed normal jugular vein flow. The shunt volume measurement by Doppler sonography showed a flow volume of 2.5L/min.

## Discussion

Patients who are hemodialysis-dependent require acute (acute renal failure) or permanent (planned initiation and continuation of dialysis) vascular access to their circulation. The choice of access type should take into account the severity of the illness (acute or chronic initiation of dialysis), the vessel anatomy, and the complication rate of the chosen access type. Three principal forms of vascular access are now available: arteriovenous autogenous access, non-autogenous (prosthetic or biograft) access, and non-tunneled or tunneled cuffed catheters. Based on American [4] and European guidelines [5], autogenous access should be preferred over non-autogenous access, which in turn should be preferred over catheters. Radial-cephalic autogenous access should be the first choice for vascular access. Nevertheless, in patients without an existing or functioning arteriovenous access who require acute hemodialysis, a central vein catheter has to be placed. Usually non-tunneled double lumen catheters are used in the acute setting, whereas tunneled, cuffed single or double lumen catheters are used for intermediate and long-term vascular access. Tunneled cuffed catheters can also be an acceptable form of vascular access in patients with an expected dialysis duration of less than one year (bridge to living-related transplant, high co-morbidity) or in patients with medical contraindications (that is, their vasculature is not suited for creating an arteriovenous access) [4,6]. Non-tunneled and cuffed tunneled catheters can be placed in the jugular vein, the femoral vein or the subclavian vein. As catheters placed in the subclavian vein predispose to catheter-induced subclavian vein stenosis, the preferred site of insertion should be the internal jugular vein [7]. Complications of hemodialysis catheters include those associated with insertion, infection and thrombosis.

Central vein stenosis is primarily related to the placement of an ipsilateral central venous catheter but can also occur without a previous history of catheter placement in up to 40% of patients [1,2]. A study evaluating 69 patients undergoing percutaneous placement of tunneled right internal jugular vein catheters for hemodialysis [2] showed evidence of unexpected stenosis and/or angulation of the central veins in 42%. Patients who had previously had tunneled internal jugular catheters showed more than twice the incidence of such abnormalities than those without (15 out of 23 patients (65%) versus 14 out of 46 patients (30%); P = 0.009). However, a retrospective study investigating 57 patients on hemodialysis with central vein stenosis showed that six (10%) of these patients had never undergone central vein catheter placement before [1]. In four of these six patients, flow volume measurements of the vascular access were available and all showed high flow volumes (mean 2,347mL/min).

The incidence of central vein stenosis induced by the insertion of hemodialysis catheters varies with the vessel used for insertion, the population studied and the type of catheter [8]. A study using venous angiography in 100 patients on dialysis (50% subclavian catheters and 50% jugular vein catheters) showed central vein stenosis in 42% of patients in the subclavian group and in 10% of patients in the jugular vein group. In pathophysiological terms, early intimal injury associated with focal endothelial denudation occurs in vessels after the insertion of central vein catheters [3]. With a longer duration of catheter use, thickening of the vein wall, enhanced smooth muscle cell production and catheter attachment to the vessel wall with thrombus and collagen formation take place. Thus, subsequent narrowing of vessel lumina is supposed to be primarily related to endothelial injury and mechanical irritation [9].

The management of central vein stenosis ranges from a conservative approach, such as clinical observation of the patient, to radiological intervention or surgery. An asymptomatic stenosis, even if greater than 50%, generally does not require treatment. This was demonstrated in a retrospective study of 35 patients on hemodialysis with asymptomatic central vein stenosis. The lesions were left untreated in 28% and none of them progressed to symptomatic stenosis. The remaining lesions were treated with angioplasty and 8% of these patients developed symptomatic stenosis [10]. If treatment of a symptomatic central vein stenosis is required, percutaneous intervention with transluminal angioplasty is the treatment of choice as the surgical therapy of these central lesions remains very complex. This approach is in consent with the Kidney Disease Outcomes Quality Initiative guidelines for vascular access [4]. These guidelines recommend that stent placement should be performed in cases of an acute elastic recoil of the vein (greater than 50% stenosis) after angioplasty, or in patients with recurrent stenosis within a three-month period post-angioplasty.

Hemodialysis vascular accesses with high flow volumes predispose to a high rate of recurrence after interventional treatment. Recent studies have shown that vascular access flow reduction by banding of the access inflow reduced the rate of re-stenosis and was able to resolve symptoms associated with non-correctable central venous lesions in patients who had previously undergone angioplasty and stent placement [11,12]. The possibility of vascular access flow reduction was discussed with our patient and vascular surgeons, but because of the primary success of the interventional treatment, we decided to keep this alternative in case of a recurrence of central vein stenosis.

## Conclusion

Our patient presented with a symptomatic near-total central vein stenosis and was successfully treated with balloon angioplasty followed by stent placement. Central vein stenosis is not a rare problem in patients on hemodialysis and can even occur without previous placement of central venous catheters. High shunt volumes seem to increase the risks associated with central vein catheters.

## Consent

Written informed consent was obtained from the patient for publication of this manuscript and any accompanying images. A copy of the written consent is available for review by the Editor-in-Chief of this journal.

## Competing interests

The authors declare that they have no competing interests.

## Authors’ contributions

UG and RB wrote the manuscript. MS performed the computed tomography venography and interventional treatment. UG and KH performed duplex sonography. KK, UB and UG were the primary clinical physicians for our patient. MS and BK revised the manuscript critically for important intellectual content. All authors read and approved the final manuscript.
